# The Influence of the “Straighten Your Back” Command on the Sagittal Spinal Curvatures in Children with Generalized Joint Hypermobility

**DOI:** 10.1155/2017/9724021

**Published:** 2017-01-01

**Authors:** Dariusz Czaprowski, Paulina Pawłowska, Aleksandra Kolwicz-Gańko, Dominik Sitarski, Agnieszka Kędra

**Affiliations:** ^1^Department of Physiotherapy, Józef Rusiecki University College, Bydgoska 33, 10-243 Olsztyn, Poland; ^2^Department of Posture Correction and Compensation, Faculty of Physical Education and Sport in Biala Podlaska, Józef Piłsudski University of Physical Education in Warsaw, Akademicka 2, 21-500 Biala Podlaska, Poland

## Abstract

*Objectives*. The aim of the study was to assess the change of sagittal spinal curvatures in children with generalized joint hypermobility (GJH) instructed with “straighten your back” command (SYB).* Methods*. The study included 56 children with GJH. The control group consisted of 193 children. Sacral slope (SS), lumbar lordosis (LL), global thoracic kyphosis (TK), lower thoracic kyphosis (LK), and upper thoracic kyphosis (UK) were assessed with Saunders inclinometer both in spontaneous positions (standing and sitting) and after the SYB.* Results*. Children with GJH after SYB presented the following: in standing, increase in SS and decrease in TK, LK, and UK (*P* < 0.01), with LL not significantly changed; in sitting: decrease in global thoracic kyphosis (35.5° (SD 20.5) versus 21.0° (SD 15.5), *P* < 0.001) below the standards proposed in the literature (30–40°) and flattening of its lower part (*P* < 0.001). The same changes were observed in the control group.* Conclusions*. In children with generalized joint hypermobility, the “straighten your back” command leads to excessive reduction of the global thoracic kyphosis and flattening of its lower part. Therefore, the “straighten your back” command should not be used to achieve the optimal standing and sitting positions.

## 1. Introduction

Generalized joint hypermobility (GJH) is diagnosed when mobility of small and large joints is increased in relation to standard mobility for any given age, gender, and race and after excluding systemic diseases [1, 2]. GJH might lead to osteoarthritis and lower back, knees, and foot pain [3, 4]. GJH is more common also in children with idiopathic scoliosis [5].

The occurrence of GJH ranges from 10 to 15% in boys and from 20 to 40% in girls [3]. It confirms the prevalence of GJH. However, while diagnosing body posture and subsequently planning the exercises, the possibility of it occurring is not usually taken into consideration and children are not differentiated in this respect [6]. It might arise from an insufficient level of physical therapists' knowledge of how to deal with hypermobile children as well as a limited number of publications on joint hypermobility in physical therapy literature [7]. The next reason may be the fact that the evaluation of musculoskeletal system based only on the examination of the flexibility of the lumbo-pelvic-hip complex muscles is not sufficient to recognize GJH [8].

Due to decreased joint stability and ligament laxity, in children with joint hypermobility, the faults of body posture are commonly recognized [4]. In consequence, they participate in various preventive and therapeutic programs [6, 8–11]. One of the ways of carrying out such programs is shaping the appropriate active self-correction [12, 13]. According to Weiss et al., being able to assume and maintain the properly corrected body posture while performing daily living activities is one of the factors which determine the efficacy of corrective exercises concerning body posture improvement [12]. Raising the awareness of the proper active self-correction is a component of conservative treatment for children with idiopathic scoliosis [13].

One of the important factors determining the quality of body posture is the spinal curvature in the sagittal plane [9, 11, 14–16]. Slight lumbar lordosis and thoracic kyphosis are expected in a perfect standing position [11, 16]. The optimal sitting position is still being discussed [16–19]. Some authors maintain that spinal curves during sitting should be similar to “ideal” standing position [16, 20].

In order to achieve a change in body posture while sitting and standing, various commands are used. In particular “straighten your back” command is commonly employed [21]. The command is utilized during physical therapy sessions as well as being the guidance given by physicians; they are also commonly used by parents and Physical Education teachers [21].

However, the influence of the command on sagittal profile of the spine was assessed only in one study, where the authors did not differentiate the children in respect of GJH occurrence. As GJH is related to a decreased ability to properly determine the angular position of joints and to a tendency to increase postural stability by assuming end-range positions of joints [3, 22], determining the influence of GJH on the magnitude of sagittal spinal curvatures and the interpretation of the command given in order to improve the body posture is interesting.

The purpose of the study was to determine the active self-correction expressed by the change in the magnitude of sagittal spinal curvatures in standing and sitting positions in children with generalized joint hypermobility instructed with “straighten your back” command.

## 2. Material and Methods

### 2.1. Subjects

The recruitment to the study took place during the presentations for parents and their children. The presentations were given in 5 randomly selected primary schools. During the meetings the aim of the study as well as the inclusion criteria were presented. The inclusion criteria were as follows: written consent of parents in which they allowed their children to participate in the program; absence of injuries or musculoskeletal pain in the previous 6 months; no neurological and systemic diseases; no previous guidance on how to adopt the corrected body posture. Overall, 450 children with parents participated in the presentations. The inclusion criteria were met by 249 children (136 girls and 113 boys) aged 10–14 years (mean 11.8, SD 0.8). The other children (*n* = 201) did not meet these criteria.

The assessment of generalized joint hypermobility (GJH) in all subjects using 9-point Beighton scale was performed by one researcher (first author) [1, 3]. The criteria for diagnosis of GJH included the score of 4 or greater [3, 4, 23, 24]. Fifty-six children met the criteria (GJH group). The remaining nonhypermobile children formed a control group (*n* = 193).

The Ethical Commission at Józef Rusiecki University College granted permission for this research (permission number: 2/2012).

### 2.2. Measurement Protocol

#### 2.2.1. Evaluation of Sagittal Spinal Curvatures

Saunders inclinometer was used to evaluate spinal curvatures in the sagittal plane (Baseline Digital Inclinometer, Saunders Group Inc., Chaska, MN, USA). The measurements were performed in accordance with the producer's instructions following the American Medical Association guidelines [25, 26]. Before taking the measurements, a nontoxic skin marker was used to mark the following points found by palpation [27]: lumbosacral junction, L5/S1 (LS point), thoracolumbar junction, T12/L1 (TL point), cervicothoracic junction, C7/T1 (CT point), and T6/T7 junction (T6 point). The sacral slope angle was measured by resetting the inclinometer in the horizontal position and placing it at the LS point. The angle of lumbar lordosis was measured after resetting the inclinometer at the LS point and placing it at the TL point. The global thoracic kyphosis angle was measured by resetting the inclinometer at the TL point and placing it at CT point. Additionally, the value of lower (T6/T7–T12/L1) and upper thoracic kyphosis (C7/T1–T6/T7) was determined. The lower thoracic kyphosis was measured by resetting the inclinometer at the TL point and placing it at T6 point. The upper thoracic kyphosis was measured by resetting the inclinometer at the T6 point and placing it at the CT point. Each measurement was taken three times, and the average value was used for analysis [25, 26]. Angles of kyphotic curves were represented as positive values, whereas lordotic curves were represented as negative values [16].

#### 2.2.2. Measurement of Sagittal Spinal Curvatures in a Standing Position

The examination was performed with subjects in a spontaneous standing position, shoeless. Their lower limbs were extended at the knee joint, with feet hip-width apart. The upper limbs were in a spontaneous position at the side of the body. The children were neither instructed on how to perform the posture correction movement nor given any feedback on their posture.

In the first phase, the magnitude of sagittal spinal curvatures was evaluated with subjects in a habitual, spontaneous standing position. Directly afterwards, the child was instructed with the “straighten your back” command and performed active self-correction. The corrected position was held for 5 seconds and the measurements were repeated.

#### 2.2.3. Measurement of Sagittal Spinal Curvatures in a Sitting Position

The examination was performed on a therapeutic table with subjects in a sitting position, without any back support. The feet were put flat on the floor. The height of the table was adapted to each child individually on the posterior knee crease level to achieve the most natural and comfortable position, with the hip and knee joints flexed at 90 degrees [17]. The angles were verified with a goniometer. Hands rested on laps. Subjects were requested to view a designated point ahead at eye level [17].

Each child was asked to assume a spontaneous, relaxed position when instructed with “sit as you usually do” command. After 5 seconds, spinal curvatures in the sagittal plane were measured. Then, the subject was given the “straighten your back” command and after 5 seconds the measurement was taken.

#### 2.2.4. Pilot Reliability Study

Before the main part of the study, the reliability of the measurements and the measurement error were determined [28, 29]. For this purpose, the evaluation of spinal curvatures was performed in 30 subjects randomly selected from the study group. The measurements were performed by one researcher (first author) on every subject twice, one week apart, in line with the methodology described above.

In the spontaneous standing position the reliability level and the measurement error were as follows: (1) for sacral slope 0.85 and 3.3°; (2) for lumbar lordosis angle 0.87 and 3.2°; (3) for global thoracic kyphosis angle 0.83 and 3.8°; (4) for lower thoracic kyphosis angle 0.82 and 3.3°; and (5) for upper thoracic kyphosis angle 0.86 and 2.8° [27, 28].

In the spontaneous sitting position the reliability level and the measurement error were as follows: (1) for sacral slope 0.89 and 2.3°; (2) for lumbar lordosis angle 0.99 and 2.5°; (3) for global thoracic kyphosis angle 0.91 and 1.9°; (4) for lower thoracic kyphosis angle 0.97 and 2.5°; and (5) for upper thoracic kyphosis angle 0.97 and 1.7°.

### 2.3. The Evaluation of Active Self-Correction after “Straighten Your Back” Command

The magnitude of sagittal spinal curvatures in spontaneous standing and sitting positions was compared with positions adopted after the “straighten your back” command. The comparison was made in children with GJH as well as in a control group, separately. Next, the magnitude of sagittal spinal curvatures adopted in GJH subjects instructed with the “straighten your back” command was compared with the parameters obtained in the control group.

### 2.4. Statistical Analysis

Statistical analysis was performed with Statistica 7.1 (StatSoft, Poland). The Shapiro-Wilk test was applied to analyze the normal distribution. Descriptive statistics, means and standard deviations, median, and quartile range were calculated for study variables for children with and without GJH. Wilcoxon test was used to estimate the difference between a spontaneous and a corrected posture assumed by children after the “straighten your back” command. Independent* t*-test and the Mann–Whitney* U* test were used to evaluate the differences between parameters: age, height, weight, BMI, and sagittal curvatures of the spine in spontaneous and corrected posture in both standing and sitting in GJH versus control group. The value *α* < 0.05 was adopted as the level of significance while CI for estimates was 0.95%.

## 3. Results

No significant differences were found between the children from GJH group and control group in respect to age, height, weight, BMI, and the magnitude of sagittal spinal curvatures in a spontaneous standing and sitting position (apart from global thoracic kyphosis in standing and sitting positions and its lower part in a sitting position) (Table 1).

### 3.1. Children with Generalized Joint Hypermobility

In standing, a significant (*P* < 0.05) change in the magnitude of the most of the spinal parts was observed in children with GJH instructed with the “straighten your back” command. Sacral slope increased, whereas global thoracic kyphosis as well as its lower and upper parts decreased. The only part of the spine without significant change was a lumbar lordosis (*P* > 0.05). In a spontaneous sitting, kyphotic position of sacral slope and lumbar lordosis was observed which assumed a slightly lordotic position when instructed with the command. Global thoracic kyphosis decreased while its lower part flattened (Table 2).

### 3.2. Nonhypermobile Children

The same pattern of changes in sagittal spinal curvatures was observed in children without GJH after the “straighten your back” command. The only exception was lumbar lordosis in a standing position which significantly decreased (*P* = 0.003) (Table 2).

The analysis did not show significant differences (*P* > 0.05) in the magnitude of particular spinal parts achieved by children with GJH and nonhypermobile children after the “straighten your back” command (Table 3).

## 4. Discussion

The aim of the study was to assess the change of sagittal curvatures of the spine in children with GJH instructed with the “straighten your back” command.

Sagittal curvatures of the spine are one of the most important factors determining the quality of standing and sitting positions [9, 11, 16]. In particular, a sitting position is important, as nowadays sedentary lifestyle dominates in children and adolescents [9, 30]. Claus et al. enumerates four types of sitting postures: (1) slump (thoracolumbar and lumbar spine in a kyphotic position), (2) flat (thoracolumbar and lumbar spine in a vertical position), (3) long lordosis (thoracolumbar and lumbar spine in a lordotic position), and (4) short lordosis (thoracic kyphosis and lumbar lordosis). Short lordosis is proposed as “ideal” as it enables achieving proper spinal curves in standing [16].

In turn, Caneiro et al. and O'Sullivan et al. distinguished three types of sitting postures: (1) slump sitting; (2) lumbopelvic upright sitting, and (3) thoracic upright sitting [17, 18].

The long lordosis and thoracic upright sitting are regarded as nonoptimal since they approach the end-range positions and may lead to an increased activity of thoracic erector spinae at T4 level and iliocostalis longissimus pars thoracis and as a consequence might lead to the higher risk of greater stress to articular and ligamentous structures, greater compression load on cervicothoracic spine, and potential discomfort [16–19].

On the other hand, assuming a neutral spine position involving slight lumbar lordosis and a relaxed thorax is recommended as this position increases trunk muscles activity without activating large, torque-producing muscles [18, 19]. Such a position also modifies the activity of key cervicothoracic muscles which might be vital in maintaining the correct sitting posture without the excessive muscle activity [17–19].

### 4.1. Active Self-Correction in Children with Generalized Joint Hypermobility

The study revealed that in a standing position sacral slope increased, whereas the global thoracic kyphosis as well as its upper and lower parts decreased after the “straighten your back” command. The lumbar lordosis did not change significantly.

In a sitting position, the adoption of lordotic position of sacral slope and lumbar lordosis was observed. However, it should be noticed that these positions were characterized by a low angular value. After the command, the global thoracic kyphosis significantly decreased and so did its lower part which assumed median values of 1.0° (QR 12.0). It should be emphasized that the magnitude of global thoracic kyphosis after SYB was considerably below standards proposed by Anderson and Cocchiarella [25] and Saunders [26] (30°–40°). Therefore, taking into account the angular values adopted after the “straighten your back” command in the thoracic kyphosis and its lower part and an increased activity of back muscles related to them [16–19], the command should be regarded as improper for improving spontaneous sitting position.

It is also worth noting that for global thoracic kyphosis and its lower part in standing as well as sacral slope, lumbar lordosis, global thoracic kyphosis, and its lower part in sitting, the differences between particular measurements were large and considerably exceeded the magnitude of measurement error. Therefore, we believe that the main observation we made concerning the excessive reduction of global thoracic kyphosis and flattening of its lower part after the “straighten your back” command is significant and may be applied in clinical practice.

### 4.2. The Comparison of Sagittal Spinal Curvatures in Children with Generalized Joint Hypermobility and Nonhypermobile Children Adopted before and after the “Straighten Your Back” Command

#### 4.2.1. Spontaneous Positions

The study revealed that in both spontaneous standing and sitting positions no significant differences exist between children with GJH and nonhypermobile children in respect of sacral slope, lumbar lordosis, upper thoracic kyphosis, and lower thoracic kyphosis (in standing). The only differences were observed in global thoracic kyphosis (in standing and sitting) and its lower part (in sitting). The children with GJH presented larger global thoracic kyphosis in standing. In spontaneous sitting, children with GJH presented smaller global thoracic kyphosis and larger lower part of it. However, the value of these differences was smaller than the measurement error found in pilot reliability study. Therefore, from the clinical point of view, they seem to have a little clinical relevance.

In spontaneous standing, both children with and without GJH presented slight sacral slope and the appropriate (30°–40°) [25, 26] angle of lumbar lordosis. The angle of global thoracic kyphosis compared with the standards proposed in literature [25, 26] was slightly too large.

In spontaneous sitting, the children from both groups adopted a kyphotic position of the whole spine. According to Murray, the poor sitting posture is typical of children with joint hypermobility [4]. However, our study revealed no significant differences between nonhypermobile children and children with GJH in respect of sagittal profile of the spine. Therefore, it seems that slump sitting is not connected with GJH but is rather typical for most children.

#### 4.2.2. Positions Adopted after “Straighten Your Back” Command

To the best of the authors' knowledge, only one study investigating the influence of the “straighten your back” command on sagittal spinal curvatures has been carried out so far. However, in the previous research, the same group of authors did not differentiate the children in respect of the occurrence of GJH [21]. Yet, taking into account the fact that those children are diagnosed with proprioceptive disorders, which leads to limited ability to determine the angular position of joints [3, 22], it is reasonable and important to analyze whether the interpretation of the “straighten your back” command will be the same in children with and without GJH.

Our research revealed no significant differences between the values of particular spinal segments adopted by children with and without GJH after the “straighten your back” command. After “straighten your back” command, the children with GJH demonstrated similar changes in spinal curvatures to those observed in children without joint hypermobility. The only difference concerned the lack of significant change in lumbar lordosis in standing. In our opinion, it proves that the children with and without GJH perform active self-correction after the command in the same way.

### 4.3. Practical Relevance

The results of the study revealed that children with GJH who were not provided with any guidance were not able to acquire the global thoracic kyphosis in sitting in accordance with the standards proposed in the literature [25, 26] when instructed with the “straighten your back” command.

It should be noted that postures adopted after the given command were characterized by the flattening of lower thoracic kyphosis which may mean moving further from mid-range and as a consequence moving towards end-range of motion. Moreover, lower thoracic kyphosis plays a key role in maintaining rotational stabilization of the spine and its lordotic position is typical for progressive idiopathic scoliosis [31].

Therefore, the flat alignment of lower thoracic kyphosis adopted after SYB should be assessed as not optimal. In consequence, the command ought not to be used to achieve the optimal sitting positions during physical therapy sessions or Physical Education lessons or at home, and it is essential to supplement exercise programs with the education aimed at explaining the proper pattern of posture correction.

Russek also emphasizes that education is one of the major methods of treatment that physical therapists can employ while dealing with children with joint hypermobility [32]. It is all the more important as the majority of people are unable to adopt short lordosis curves without facilitation and feedback and if the correction is made without a therapist's assistance it is made by extension of the thoracic spine [16]. It is also confirmed by Claus et al. who claim that the adoption of a proper spine posture may be difficult [16].

### 4.4. Limitations

The current study covered children. Therefore, it is worth noting that the majority of studies concerning various standing and sitting postures focus on adults [16–18, 33]. For that reason, caution is advised while comparing the results of this study with the results presented by other authors without previously comparing active self-correction between children and adults. However, we believe that it is essential that children are included in the present study since they are referred to various therapeutic and preventive programs [6, 7, 9–11].

Despite certain limitations we believe that the results of this study may have relevance to the scientific and clinical approach since the study covered a wide, homogenous group and it was conducted using reliable diagnostic tools [3, 23, 25, 26]. Additionally, the reliability of the measurements ranged from excellent to good [28, 29]. Therefore, the results of this study may be considered to be practically important.

The obtained results constitute another element of the debate about the possibility of executing the most desired standing and sitting postures as well as increasing the effectiveness of therapeutic programs which children with spinal deformities and back pain are covered by. It seems that, in future studies, it is also important to evaluate the active self-correction, after teaching the children how to perform it.

## 5. Conclusions

Children with generalized joint hypermobility perform a similar corrective movement after the “straighten your back” command to children without GJH. The command leads to the excessive reduction of the global thoracic kyphosis as well as flattening of its lower part in both standing and sitting positions. Therefore, this command should not be used to achieve the optimal standing and sitting.

## Figures and Tables

**Table 1:** The comparison of age, height, weight, BMI, and sagittal spinal curvatures in spontaneous standing (St) and sitting positions (Si) between children with generalized joint hypermobility (GJH) and control group (control).

	GJH	Control	*P* value
	*n* = 56	*n* = 193	
Age (years)	12.0 (1.0)	12.0 (1.0)	0.31^*∗*^
Weight (kg)	42.8 (18.5)	41.5 (14.0)	0.72^*∗*^
Height (cm)	151.7 (8.2)	151.0 (8.4)	0.84^†^
BMI (kg m^−2^)	18.9 (5.9)	18.1 (4.4)	0.83^*∗*^
SS St (°)	−18.9 (6.7)	−19.5 (6.3)	0.5^†^
LL St (°)	−32.3 (9.2)	−33.2 (8.6)	0.5^†^
TK St (°)	43.7 (11.0)	42.4 (8.8)	**0.03** ^†^
LK St (°)	11.1 (7.6)	9.1 (7.7)	0.9^†^
UK St (°)	33.0 (7.7)	33.4 (7.4)	0.7^†^
SS Si (°)	13.0 (11.5)	12.0 (10.0)	0.9^*∗*^
LL Si (°)	18.5 (16.0)	18.0 (12.0)	0.6^*∗*^
TK Si (°)	36.0 (13.0)	36.7 (10.1)	**0.01** ^†^
LK Si (°)	15.7 (10.3)	15.1 (8.2)	**0.02** ^†^
UK Si (°)	21.0 (10.5)	21.0 (12.0)	0.3^*∗*^

Statistically significant differences are in bold; ^†^values are in mean (SD) and by independent *t*-test; ^*∗*^values are in median (QR) and by Mann–Whitney *U* test.

SS: sacral slope; LL: lumbar lordosis; TK: global thoracic kyphosis; LK: lower thoracic kyphosis; UK: upper thoracic kyphosis.

**Table 2:** The comparison of magnitude of sagittal spinal curvatures in children with generalized joint hypermobility (GJH) and control group (control) in a spontaneous posture (SP) and actively self-corrected (ASC) standing (St) and sitting (Si) positions.

		GJH			Control	
	SP	ASC	*P* value	SP	ASC	*P* value
	Median (QR)	Median (QR)		Median (QR)	Median (QR)	
SS St (°)	−18.5 (9.5)	−19.0 (9.0)	0.006^*∗*^	−20.0 (7.0)	−21.0 (9.0)	<0.001^*∗*^
LL St (°)	−31.0 (13.0)	−30.5 (11.5)	0.6^*∗*^	−34.0 (11.0)	−31.0 (14.0)	0.003^*∗*^
TK St (°)	42.0 (15.5)	34.5 (16.0)	<0.001^*∗*^	43.0 (13.0)	33.0 (15.0)	<0.001^*∗*^
LK St (°)	10.5 (11.0)	5.0 (14.0)	<0.001^*∗*^	9.0 (9.0)	3.0 (9.0)	<0.001^*∗*^
UK St (°)	34.5 (9.0)	31.0 (11.5)	0.002^*∗*^	32.0 (10.0)	31.0 (13.0)	<0.001^*∗*^
SS Si (°)	13.0 (11.5)	−8.0 (12.5)	<0.001^*∗*^	12.0 (10.0)	−8.0 (11.0)	<0.001^*∗*^
LL Si (°)	18.5 (16.0)	−6.0 (14.0)	<0.001^*∗*^	18.0 (12.0)	−7.0 (14.0)	<0.001^*∗*^
TK Si (°)	35.5 (20.5)	21.0 (15.5)	<0.001^*∗*^	37.0 (14.0)	23.0 (17.0)	<0.001^*∗*^
LK Si (°)	16.5 (17.0)	1.0 (12.0)	<0.001^*∗*^	16.0 (12.0)	1.0 (11.0)	<0.001^*∗*^
UK Si (°)	21.0 (10.5)	22.0 (12.0)	0.02^*∗*^	21.0 (12.0)	23.0 (12.0)	0.001^*∗*^

Statistically significant differences are in bold; ^*∗*^values are in median (QR) and by Wilcoxon test.

SS: sacral slope; LL: lumbar lordosis; TK: global thoracic kyphosis; LK: lower thoracic kyphosis; UK: upper thoracic kyphosis.

**Table 3:** Comparison of magnitude of sagittal spinal curvatures between children with generalized joint hypermobility (GJH) and control group (control) in actively self-corrected (ASC) standing (St) and sitting (Si) positions.

		ASC			ASC	
		St			Si	
	GJH	Control	*P* value	GJH	Control	*P* value
SS (°)	−19.0 (9.0)	−21.0 (9.0)	0.3^*∗*^	−6.7 (8.3)	−7.2 (8.2)	0.9^†^
LL (°)	−31.9 (10.2)	−31.2 (10.4)	0.9^†^	−5.5 (10.2)	−7.0 (10.6)	0.8^†^
TK (°)	33.7 (12.9)	33.1 (11.3)	0.2^†^	21.0 (15.5)	23.0 (17.0)	0.5^*∗*^
LK (°)	5.0 (14.0)	3.0 (9.0)	0.1^*∗*^	0.8 (8.7)	1.0 (8.4)	0.7^†^
UK (°)	31.0 (11.5)	31.0 (13.0)	0.9^*∗*^	22.0 (12.0)	23.0 (12.0)	0.5^*∗*^

^†^Values are in mean (SD) and by independent *t*-test; ^*∗*^values are in median (QR) and by Mann–Whitney *U* test.

SS: sacral slope; LL: lumbar lordosis; TK: global thoracic kyphosis; LK: lower thoracic kyphosis; UK: upper thoracic kyphosis.
